# Early Life and Nutrition and Allergy Development

**DOI:** 10.3390/nu14020282

**Published:** 2022-01-11

**Authors:** Yvan Vandenplas

**Affiliations:** KidZ Health Castle, UZ Brussel, Vrije Universiteit Brussel, 1090 Brussels, Belgium; Yvan.Vandenplas@uzbrussel.be

Much evidence has been accumulated over recent years on the importance of the first 1000 days of a child’s life, starting from conception to the postnatal age of two years, with regard to the risk of developing allergic disease. This relatively short timeframe has life-long effects on the risk of the individual developing immune-mediated diseases such as asthma, diabetes, inflammatory bowel disease, among others. Evidence from epidemiological studies showed an important increase in both the incidence and prevalence of allergic diseases. The importance of the genetic background, i.e., a family history of allergic disease, has been known for many years. Major contributing environmental factors include pollution, changing living conditions, nutrition and exposure to medications. The influences of these environmental factors are thought to be mediated by epigenetic mechanisms, which are heritable, reversible, and biologically relevant biochemical modifications of the chromatin carrying the genetic information without changing the nucleotide sequence of the genome [1]. The genotype is known to determine, at least in part, the impact of environmental endotoxin [2]. Human milk is one of the richest sources of miRNAs, which are one of the important epigenetic mechanisms underlying the beneficial effects of human milk in breastfed infants [1]. Yet, a beneficial effect of breastfeeding on allergic disease in comparison to non-breastfed infants could not be demonstrated [3,4].

Multiple factors interfering with the development of allergic disease contribute to the contradictions between the probability of allergy prevention between the outcomes of animal trials and real-life studies. Confounding variables can be controlled in a laboratory set-up. Partial hydrolysates were shown, in laboratory conditions, to differ in peptide size, residual allergenicity and the development of tolerance [5,6]. Compared to intact protein, partially hydrolyzed whey proteins represent a significantly lower “allergenic load”, reduced by a factor of at least 1000, contain bioactive peptides and modulate the immune system [5,6]. Partial hydrolysate sensitization did not induce whey-induced clinical symptoms, even though sensitization was established [7]. Increased regulatory cell populations in the systemic immune system and the prevention of increased total Th1 and activated Th17 in the intestinal immune organs could contribute to the suppression of allergic symptoms [7]. Sensitization can also occur through skin contact with cow’s milk protein [8].

The early-life composition of the gastro-intestinal (GI) microbiota is another factor of major importance influencing the risk of developing allergic disease. A bifidogenic GI microbiome may decrease the risk of developing allergic disease [9]. The role of the birth mode as a risk factor for allergic disease is debated and may depend on the diversity of the GI microbiota [10,11]. In a longitudinal study of the fecal microbiota of children from 5 weeks through 6 to 11 years, changes in the diversity and composition of the GI microbiota were associated with the development of allergies and asthma [11]. Multiple animal and human studies report a positive association between pre- and perinatal antibiotic use during pregnancy and disease in the offspring; however, no study on humans suggests causality [12]. Most infants born through cesarean section are also exposed to the perinatal administration of antibiotics.

The existence of a window of opportunity—or window of increased risk—is illustrated by the findings on allergy prevention. Giving complementary bottles at maternity hospitals to newborns who will be exclusively breastfed increases the risk of developing CMA later [13]. Similarly, the avoidance of dairy products during pregnancy or breastfeeding should be discouraged [13]. The European Academy of Allergy and Clinical Immunology (EAACI) concluded in 2021 that there was no recommendation for or against the use of vitamin supplements, fish oil, prebiotics, probiotics or synbiotics in pregnancy, when breastfeeding or in infancy; altering the duration of exclusive breastfeeding; and hydrolyzed infant formulas, regular cow’s milk-based infant formula after a week of age or use of emollients [14]. Intact cow’s milk protein should be avoided during the first week of life [14]. However, a major difference in the design of clinical trials with partial hydrolysates in the prevention of allergy is the (maximal) age of randomization. While many studies only consider randomization at birth, others allow randomization up to the age of one month [15].

Healthcare professionals should be educated on the important role of dietary choices in modulating gut microbiota for both preventive and therapeutically purposes. The maintenance of a healthy gut microbiota, via nutrition and the use of food supplements, could lead to the reduction in the metabolic risk associated with dangerous lifestyles. In well-controlled in vitro or animal trials, the importance of feeding and the GI microbiome is well established, indicating a beneficial effect of a diverse and bifidogenic microbiome and the protective effect of hydrolyzed protein. However, due to a multitude of confounding variables which cannot be controlled in human studies, real-life trials do not confirm the evidence from the animal and in vitro trials. As a consequence, opinions have evolved in opposite directions. One can conclude that diet during early life does not really matter since the evidence from real-life studies is not convincing. However, the absence of evidence of a benefit does not mean there is no benefit. Therefore, one can also conclude that the evidence of a benefit from well-controlled animal and in vitro studies suggests a benefit for real-life situations. Therefore, since adverse effects are not reported, one might also consider that there is evidence that feeding with hydrolyzed protein is not harmful and that some benefits cannot be excluded. As a consequence, no recommendation “for or against” can be made, and the healthcare provider should discuss the pros and cons of offering feeding with hydrolyzed protein with the care-givers. It might be that many small irrelevant effects may at the end result in a relevant change. However, clinical trials are needed to confirm this hypothesis.
